# Neoantigens elicit T cell responses in breast cancer

**DOI:** 10.1038/s41598-021-91358-1

**Published:** 2021-06-30

**Authors:** Takafumi Morisaki, Makoto Kubo, Masayo Umebayashi, Poh Yin Yew, Sachiko Yoshimura, Jae-Hyun Park, Kazuma Kiyotani, Masaya Kai, Mai Yamada, Yoshinao Oda, Yusuke Nakamura, Takashi Morisaki, Masafumi Nakamura

**Affiliations:** 1 Department of Surgery and Oncology, Graduate School of Medical Sciences, Kyushu University, 3-1-1 Maidashi, Higashi-ku, Fukuoka, 812-8582 Japan; 2 Fukuoka General Cancer Clinic, Fukuoka, Japan; 3 Cancer Precision Medicine, Inc, Kawasaki, Kanagawa Japan; 4 Department of Anatomic Pathology, Graduate School of Medical Sciences, Kyushu University, Fukuoka, Japan; 5 Cancer Precision Medicine Center, Japanese Foundation for Cancer Research, Tokyo, Japan

**Keywords:** Cancer, Computational biology and bioinformatics, Immunology, Oncology

## Abstract

Neoantigens are tumour-specific antigens that arise from non-synonymous mutations in tumour cells. However, their effect on immune responses in the tumour microenvironment remains unclear in breast cancer. We performed whole exome and RNA sequencing of 31 fresh breast cancer tissues and neoantigen prediction from non-synonymous single nucleotide variants (nsSNVs) among exonic mutations. Neoantigen profiles were determined by predictive HLA binding affinity (IC_50_ < 500 nM) and mRNA expression with a read count of ≥ 1. We evaluated the association between neoantigen load and expression levels of immune-related genes. Moreover, using primary tumour cells established from pleural fluid of a breast cancer patient with carcinomatous pleurisy, we induced cytotoxic T lymphocytes (CTLs) by coculturing neoantigen peptide-pulsed dendritic cells (DCs) with autologous peripheral lymphocytes. The functions of CTLs were examined by cytotoxicity and IFN-γ ELISpot assays. Neoantigen load ranged from 6 to 440 (mean, 95) and was positively correlated to the total number of nsSNVs. Although no associations between neoantigen load and mRNA expression of T cell markers were observed, the coculture of neoantigen-pulsed DCs and lymphocytes successfully induced CTLs ex vivo. These results suggest that neoantigen analysis may have utility in developing strategies to elicit T cell responses.

## Introduction

Treatment of breast cancer has advanced significantly in recent decades because of the development of new drug therapies^1,2^. However, many breast cancer patients still die, especially from drug resistance^3,4^. Therefore, the development of new therapeutic strategies for breast cancer is critical.

Recently, immune checkpoint inhibitors (ICIs) have shown anti-tumour efficacy in many cancer types^5–7^. Although the response rate to ICIs is limited and ICIs have been associated with immune-related adverse events, the response to ICIs is sometimes durable^8,9^. Notably, the existence of tumour antigen-specific T lymphocytes is an important factor for ICIs to be effective^10,11^. Among tumour antigens, peptides that contain amino acid substitutions caused by somatic non-synonymous mutations in tumour cells, but not in non-tumour cells, are called neoantigens.

A high number of neoantigens is expected to elicit a strong immune reaction in the tumour microenvironment. A recent study of multiple cancer types including breast cancer showed that increased numbers of neoantigens are associated with higher *CD8A* expression^12^. Another study showed that the number of neoantigens has a positive correlation to cytolytic activity in breast cancer, which is based on transcription levels of *GZMA* and *PRF1*^13^. Conversely, other studies have shown a negative effect of high neoantigen load on the immune response in breast cancer^14,15^. Therefore, the effect of neoantigen burden on the immune response in breast cancer remains controversial.

Current research has focused on developing treatments that target neoantigens^16,17^. A recent study has reported the remarkable effect of adaptive transfer of neoantigen-specific T cells in a hormone-positive metastatic breast cancer patient^18^. Another study has demonstrated that neoantigens induce anti-tumour immunity in xenograft models^19^. However, studies on the therapeutic application of neoantigens in breast cancer remain limited and further evidence is required.

In the current study, we performed neoantigen profiling of fresh tumour tissues from 31 breast cancer patients and examined the correlation between neoantigen load and the immune environment in breast cancer. We also examined whether dendritic cells (DCs) pulsed with in silico-predicted neoantigen peptides induce tumour-specific T lymphocytes in an ex vivo model.

## Results

### Mutational landscape of breast cancer patients

**Figure 1:**
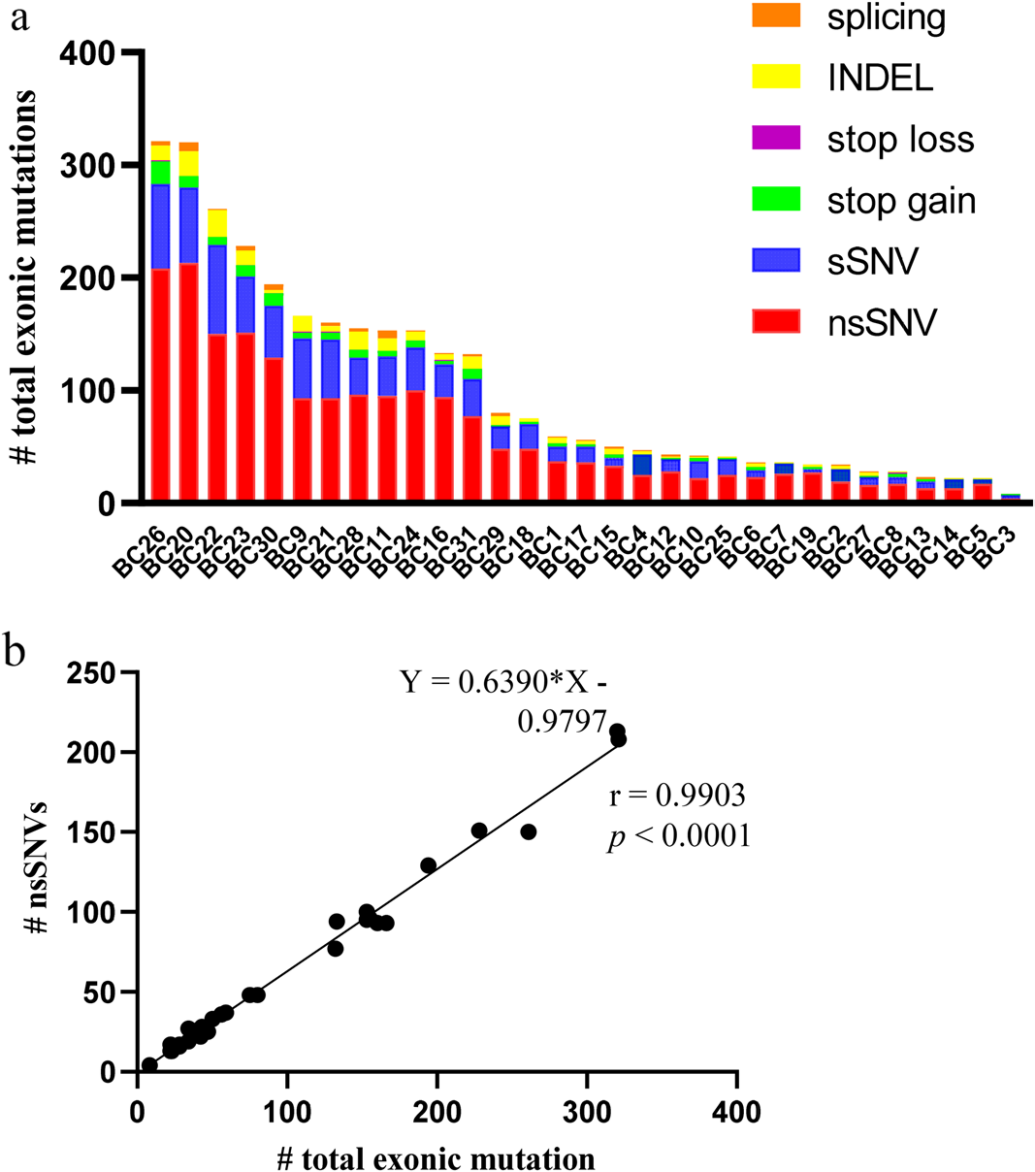
Exonic mutation landscape in breast cancer determined by whole exome sequencing of tumour tissues and peripheral blood mononuclear cells. (**a**) The numbers of total exonic mutations and mutation types in the 31 breast cancer patients. (**b**) The number of total exonic mutations was highly correlated to the number of nsSNVs. INDEL: insertion/deletion; sSNV: synonymous single nucleotide variant; nsSNV nonsynonymous single nucleotide variant.

To perform neoantigen prediction, we first detected tumour-specific gene mutations by whole exome sequencing of 31 fresh breast cancer tissues and paired samples of peripheral blood mononuclear cells (PBMCs). The clinical characteristics of the 31 breast cancer patients are shown in Table 1. We performed neoantigen prediction only on nsSNVs among exonic mutations. A total of 1976 nsSNV mutations were found in the samples from the 31 patients and nsSNVs accounted for approximately 62.4% of the total exonic mutations (Fig. 1a), which showed a strong linear correlation with total exonic mutations (r = 0.99, *p* < 0.0001, Fig. 1b). The top three most frequent genes with nsSNVs were *TP53* (13 cases), *PIK3CA* (5 cases), and *TTN* (5 cases), and five identical paired nsSNVs (*AKT1*, *ARAP3*, *NOTCH3*, *PIK3CA*, and *SLC35E2*) were identified (Table 2).

**Table 1:** Clinical characteristics of the breast cancer patients.

Patient ID	Age	Subtype	Location of tumor specimen	Prior therapy	Metastasis	Stage	Timing of biopsy since diagnosis (year)
BC1	75	Luminal A	Breast	ET, CT, RT	BONE, lymph node	IV	3
BC2	41	Luminal A	Breast	ET, CT	Bone, lymph node	IV	4
BC3	52	Luminal A	Breast	–	–	I	6
BC4	61	Luminal A	Abdominal wall	OP, ET, CT, RT	Breast,, liver, lung, bone	IV	6
BC5	49	Luminal A	Breast	–	Lymph node	IIIa	0
BC6	46	Luminal A	Breast	–	–	I	0
BC7	56	Luminal A	Liver	OP, ET, CT, RT	Liver, abdominal wall	IV	14
BC8	53	Luminal A	Breast	–	Lymph node	II	0
BC9	50	Luminal B	Lung	OP, ET, RT	Breast, lung	IV	14
BC10	45	Luminal B	Liver	OP, ET, CT, RT	Liver	IV	3
BC11	56	Luminal B	Breast	ET, CT	Pleura	IV	2
BC12	54	Luminal B	Bone	OP, ET, CT	Lung, bone	IV	8
BC13	33	Luminal B	Lung	CT	Lung	IV	2
BC14	51	Luminal B	Breast	–	Lymph node	II	0
BC15	62	Luminal B	Liver	OP, ET, CT, RT	Liver, bone	IV	3
BC16	50	Luminal HER2	Lymphnode	OP, ET, CT	Pleura, lymph node	IV	10
BC17	47	Luminal HER2	Breast	–	Lymph node	IIIb	0
BC18	46	Her2	Liver	CT, RT	Brain, bone, chest wall	IV	6
BC19	36	TNBC	Lung	OP, CT, RT	Breast, lung	IV	2
BC20	79	TNBC	Liver	OP, CT	Skin, abdominal wall	IV	9
BC21	39	TNBC	Liver	OP, CT	Liver	IV	2
BC22	32	TNBC	Breast	OP, CT	Breast, lymph node	IV	2
BC23	40	TNBC	Lymphnode	OP, CT	Lymph node	IV	3
BC24	64	TNBC	Lung	OP, ET	Lung	IV	2
BC25	39	TNBC	Breast	OP, CT, RT	Lymph node, skin	IV	1
BC26	64	TNBC	Breast	OP, CT	Chest wall	IV	13
BC27	48	TNBC	Breast	–	Lymph node	II	0
BC28	65	TNBC	Breast	–	–	II	0
BC29	42	TNBC	Liver	–	Lung	IV	0
BC30	41	TNBC	Breast	OP, CT, RT	Brain, bone, lymph node, liver	IV	2
BC31	51	TNBC	Breast	–	Lymph node	II	0

Luminal A: luminal A type, Luminal B: luminal B type, HER2: HER2-positive type, TNBC: triple negative breast cancer, OP: operation, ET: endocrine therapy, CT: chemotherapy, RT: radiation therapy.

**Table 2:** Frequently mutated genes and genes that harboured identical nsSNVs.

Patient ID	TP53 nsSNV	PIK3CA nsSNV	TTN nsSNV	Gene which harbor identical nsSNV
BC1				AKT1
BC2				
BC3				
BC4				
BC5				
BC6		〇		
BC7				
BC8	〇			
BC9	〇		〇	
BC10				
BC11	〇			ARAP3
BC12			〇	
BC13				
BC14				AKT1
BC15		〇		
BC16				
BC17		〇		SLC35E2, PIK3CA
BC18				NOTCH3
BC19	〇			
BC20			〇	
BC21	〇			SLC35E2
BC22	〇			
BC23	〇			
BC24				ARAP3
BC25	〇			
BC26	〇		〇	
BC27	〇			
BC28	〇	〇	〇	
BC29				
BC30	〇			
BC31	〇	〇		PIK3CA, NOTCH3

○: Patient with mutation. nsSNV: nonsynonymous single nucleotide variation.

### Neoantigen landscape of breast cancer patients

**Figure 2:**
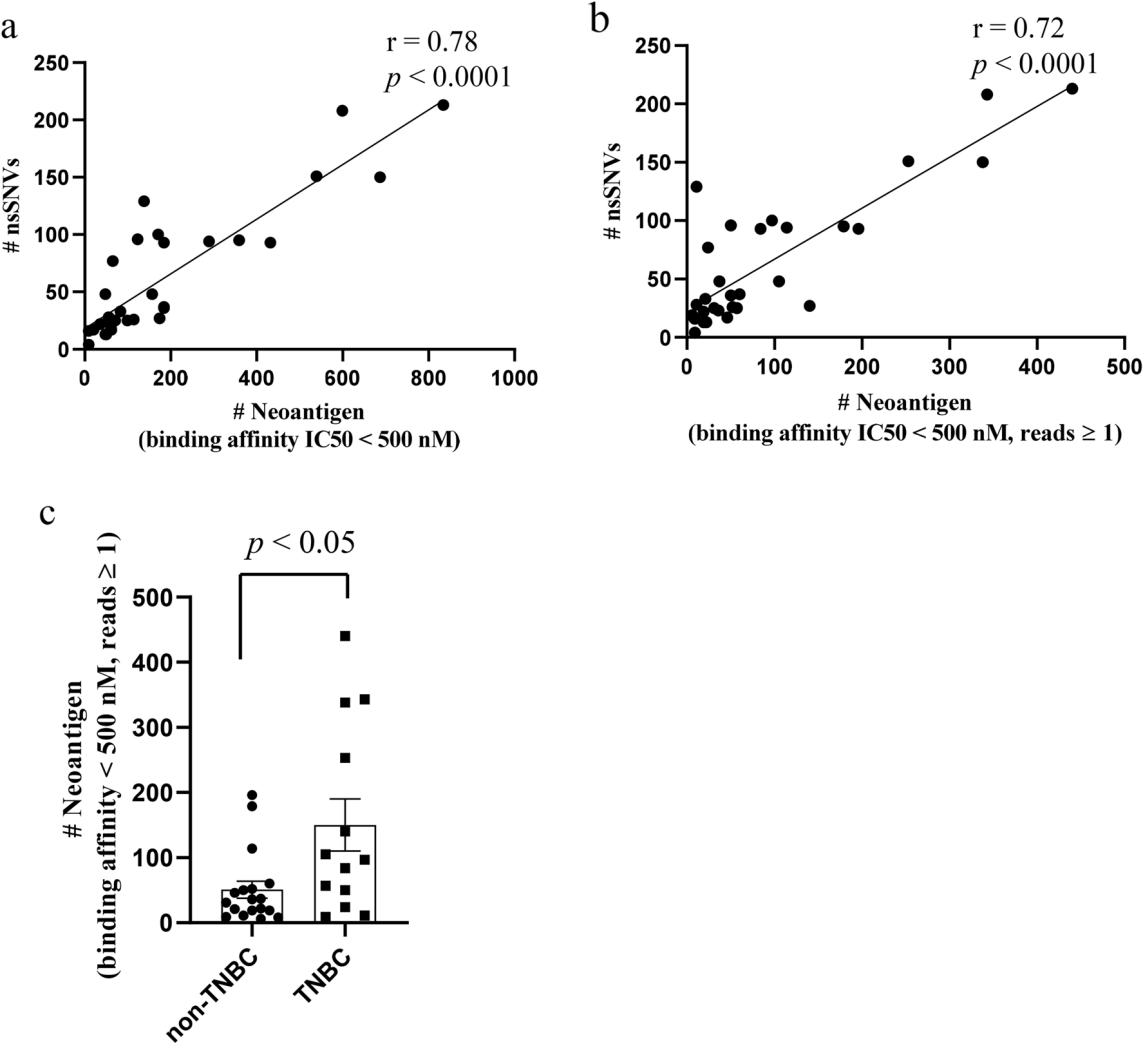
Neoantigen landscape in breast cancer determined by a computational HLA class I-binding assay and RNA sequencing. (**a**) The number of predicted neoantigens (IC50 < 500 nM) was highly correlated to the number of nsSNVs (r = 0.78, *p* < 0.0001). (**b**) The number of predicted neoantigens (IC50 < 500 nM, read count ≥ 1) was highly correlated to the number of nsSNVs (r = 0.72, *p* < 0.0001). (**c**) The number of predicted neoantigens (IC50 < 500 nM, read count ≥ 1) in TNBC was statistically higher than that in non-TNBC. TNBC: triple negative breast cancer.

We created neoantigen profiles by predicting the binding affinity of possible 8–11mer amino acid sequences generated by each nsSNV to patient-specific HLA class I molecules. The number of neoantigen peptides with a binding affinity of IC_50_ < 500 nM ranged from 9 to 834 (mean, 192), which showed a linear correlation to the number of nsSNVs (r = 0.78, *p* < 0.0001, Fig. 2a). By considering the importance of endogenous neoantigen peptides to elicit immune reactions in vivo, we added the mRNA read count, which covered the mutated site, to these criteria with a read count of ≥ 1. We refer to the number of neoantigens detected by these criteria as “neoantigen load.” The neoantigen load ranged from 6 to 440 (mean, 95) and showed a high linear correlation to the number of nsSNVs (r = 0.725, *p* < 0.0001, Fig. 2b). The neoantigen load was significantly higher in TNBC cases (n = 13; median = 150, range: 9–440) than in non-TNBC cases (n = 18; median = 51, range: 6–196) (*p* < 0.05, Fig. 2c).

### Association between the number of neoantigen peptides and immune-related gene expression

To investigate the effect of neoantigen load on the immune environment in breast cancer, the expression of immune-related genes was analysed by RNA-seq using the same sample that had been subjected to neoantigen analysis. There was no significant association between mRNA expression levels of T cell markers *CD8a*, *CD4*, and *TCRB* and the total number of nsSNVs and neoantigen load (Additional file 1). Expression levels of *GZMA*, *GZMB* and *PRF1*, which encode T cell activation markers, also did not show significant correlations (Additional file 1).

### Induction of CTLs by synthesized neoantigen peptides

**Figure 3:**
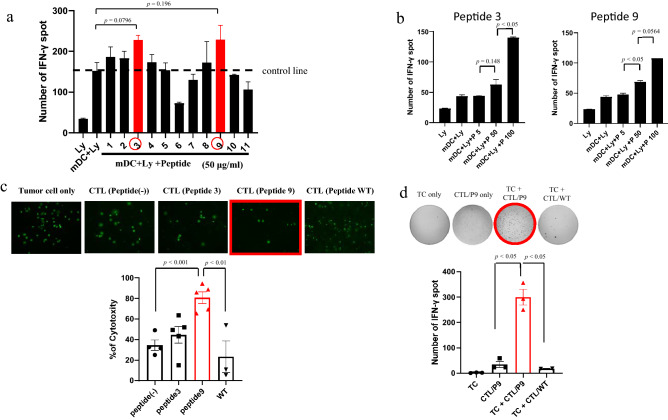
Response of cytotoxic T lymphocytes stimulated by neoantigen peptide-pulsed mature dendritic cells (mDCs). (**a**) IFN-γ ELISpot response of peripheral blood lymphocytes stimulated by neoantigen peptide-pulsed mDCs. The final concentration of peptide was 50 µg/ml. The mean number of IFN-γ ELISpots** ± **SEM is shown (n = 2). (**b**) Dose-dependent increase in the IFN-γ ELISpot response of peripheral blood lymphocytes stimulated by peptide 3- or 9-pulsed mDCs. The final concentrations of peptides were 5, 50, and 100 µg/ml. The mean number of IFN-γ ELISpots** ± **SEM is shown (n = 2). (**c**) Cytotoxicity assay of CTLs induced by 4 weeks of coculture with neoantigen-pulsed mDCs. Numbers of calcein-positive living tumour cells were significantly decreased by co-culture with CTLs stimulated with peptide 9-pulsed DCs. The mean % of cytotoxicity ± SEM is shown (n = 3–5). (**d**) IFNγ-release responses by peptide 9-pulsed mDC stimulated with CTLs against autologous tumour cells. Ly: lymphocyte; mDC, mature dendritic cell; TC, tumour cell; CTL/P9, CTLs induced by peptide 9-pulsed DCs; CTL/WT, CTLs induced by wildtype peptide-pulsed DCs. The mean number of IFN-γ ELISpot** ± **SEM. is shown (n = 2–3).

Neoantigen profiles can be applied to therapeutic applications such as cancer vaccines. Therefore, we examined whether CTLs were induced by coculturing lymphocytes with autologous DCs pulsed with the predicted neoantigen peptides. First, we performed neoantigen analysis of primary tumour cells established from pleural fluid of a TNBC patient with carcinomatous pleurisy. We synthesized 10 mutant peptides from the neoantigen profiles, which showed high affinity for HLA-A02:06 and mRNA expression with a read count of ≥ 2 (Table 3). We also synthesized a wildtype peptide as a control. After coculturing autologous peripheral lymphocytes with synthesized peptide-pulsed mDCs for 48 h, we quantified IFNγ-producing lymphocytes by an ELISpot assay. Although the results did not show statistical significance, lymphocytes pulsed with two peptides (peptides 3 and 9) showed a > 50% higher response compared with the control without peptide pulsation (Fig. 3a). We performed the same experiments using various concentrations of the two peptides and confirmed that the IFNγ-producing reaction was increased in a concentration-dependent manner (Fig. 3b). Therefore, we speculated that naïve T lymphocytes capable of reacting with peptides 3 and 9 were already present among peripheral lymphocytes. We next cultured peripheral lymphocytes with mDCs pulsed with these two peptides under low concentrations of recombinant human IL-2 for 4 weeks to induce CTLs. CTLs generated by coculture with peptide 9-pulsed mDCs showed significantly higher cytotoxicity than the wildtype peptide control in a calcein releasing assay (*p* < 0.01, Fig. 3c). The same CTLs also showed a significantly higher IFNγ-producing ability against the autologous tumour cells than the wildtype control (*p* < 0.05, Fig. 3d).

**Table 3:**
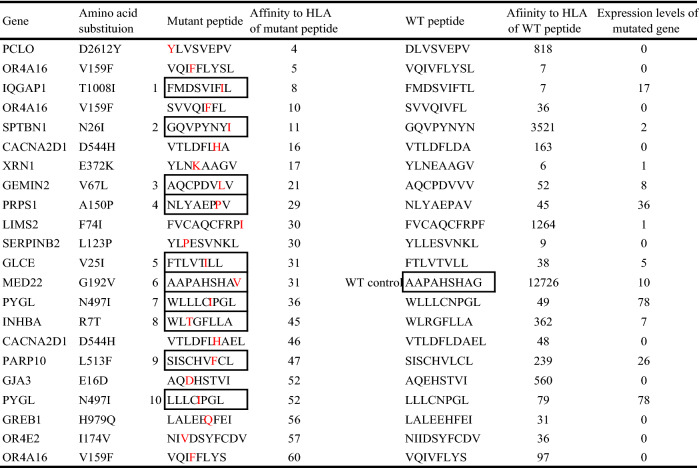
List of predicted neoantigens with a high binding affinity for HLA-A02:06.

The mutant peptides enclosed in squares indicate the candidates selected for peptide synthesis. The number on the left side of the candidate peptides refers to the synthesized peptides in the main text. WT: wildtype.

## Discussion

We conducted neoantigen profiling of 31 breast cancers using an in silico prediction pipeline and analysed the correlation of neoantigen load with immune-related gene expression. We also performed ex vivo CTL induction experiments using TNBC patient-derived primary tumour cells and autologous PBMCs along with the predicted neoantigen peptides. In this study, we obtained the following findings: (1) the neoantigen profiles were highly unique among individuals; (2) neoantigen load showed a linear correlation with the total number of nsSNVs; (3) neoantigen load was not correlated to immune-related gene expression; (4) DCs pulsed with synthetic peptides predicted from our neoantigen analysis successfully induced CTLs ex vivo*.*

In silico neoantigen prediction analysis has recently been widely performed for many cancers, which is considered to be a potentially useful tool for clinical parameters and immunotherapies^20,21^. Previous studies have shown that 8–11mer peptides with a high affinity for HLA-class I elicit CD8 + CTLs that are the most effective cells to eliminate cancer^22^. Here, we performed neoantigen profiling of 31 breast cancer specimens based on the binding affinity of possible 8–11mer peptides, which harboured substituted amino acids altered by nsSNVs, to HLA class I molecules and mRNA expression levels of mutated genes. We succeeded in detecting neoantigens in all cases, which indicated good feasibility of neoantigen analysis in breast cancer regardless of the specimen site. Among the 1976 nsSNVs detected in the 31 cases, five pairs of identical nsSNVs were observed. Of these five genes, only *AKT1* in both BC1 and BC14 remained as neoantigen candidates. Although frequently mutated genes such as *TP53*, *PIK3CA*, and *TTN* were found, there was only one pair of identical nsSNVs in *PIK3CA*. Accordingly, among the 1976 nsSNVs, only a pair of *AKT1* remained as an identical neoantigen candidate. This result is consistent with a previous report showing that the majority of neoantigens are specific to the individual^23^. Considering that most neoantigens are derived from nsSNVs, the set of neoantigens in each patient varies greatly. This result indicates that neoantigen profiling may be important in developing highly personalized therapies.

Although there are no common criteria to predict neoantigens, a binding affinity of IC_50_ < 500 nM to HLA molecules is widely used^24,25^. In this study, we used two criteria: binding affinity of IC_50_ < 500 nM and RNA-seq data with one or more read counts that contained the mutated position. Although the RNA level required to elicit a T cell reaction is unclear, a study has shown that a single peptide-MHC complex induces cytotoxic T cells, which indicates that a very low expression level may be sufficient for T cell activation^26^. Therefore, we consider that even only one read count of RNA-seq can be a marker for a neoantigen peptide to elicit a T cell response. In this study, we referred to the number of neoantigen peptides predicted by not only the binding affinity to HLA class I molecules, but also RNA expression as “neoantigen load”. Both neoantigen load and the numbers of neoantigens predicted only by binding affinity to HLA class I molecules were positively correlated to the total number of nsSNVs. This is consistent with previous TCGA-based neoantigen analyses of breast cancers^15,24^. This result suggests that tumour mutation burden (TMB) simply reflects an increased opportunity of gaining neoantigens and that TMB can be a surrogate marker for neoantigen load. In the analysis by subgroup, we found that the neoantigen load was higher in TNBC cases than non-TNBC cases, which was consistent with previous reports^15,24^. Although the mechanism underlying the higher neoantigen load in TNBC compared with non-TNBC has not been elucidated, a possible cause is that, compared with luminal type breast cancer, which has specific tumorigenic factors such as signalling pathways via hormone receptors, TNBC requires a higher rate of mutations to gain tumour-driving mutations.

In contrast to our expectation, neoantigen load did not show positive correlations with mRNA expression of T cell markers or activated T cell markers. Consistent with our results, another report revealed an inverse association between immune metagene expression and TMB or neoantigen load in TNBC and the HER2-positive type^14^. Another report also showed that a low TMB and neoantigen load were associated with high immune cell infiltration in TNBC^15^. Moreover, this trend has been reported in multiple tumour types^27^. One reason for the inability of a high neoantigen load to elicit immune-related gene expression may be the complexity of immunity in the tumour microenvironment, which includes multiple immune cell populations and cytokines^28,29^. Even if neoantigens are endogenously expressed and a immune response occurs, the existence of immune suppressive factors, such as regulatory T cells, myeloid derived suppressor cells, and immune suppressive cytokines, may counteract the immunogenicity of neoantigens. Another reason may be the neoantigen heterogeneity in breast cancers^30,31^. Several studies have demonstrated that tumour heterogeneity correlates with a reduced immune response^15,32^. Collectively, although neoantigens themselves induce immune responses, many other factors in the tumour microenvironment may cancel the immunogenicity of neoantigens.

The relationship between neoantigen load and tumour-infiltrating lymphocytes (TILs) is a very interesting research area^33^. However, in about half of the cases in the current study, we were unable to evaluate TILs because of the limited biopsy specimens that were available and prioritized them for neoantigen analysis. TIL evaluation of limited samples suggested no significant correlation between TILs and neoantigen load (data not shown). Consistent with this result, a recent study has shown no correlation between the number of nsSNVs and stromal TILs within each subtype of breast cancer^34^. We believe that the presence of neoantigen-specific TILs is more important than the number of TILs or neoantigen load and further analyses are required to address this issue.

In addition to the use of neoantigen profiles as a clinical immune parameter, there is great interest in their therapeutic use, such as for neoantigen vaccines and adaptive immunotherapy of neoantigen-specific CTLs^16,17^. Using autologous tumour cells, monocyte-derived DCs, peripheral lymphocytes, and synthetic neoantigen peptides, we were able to successfully induce CTLs ex vivo. Importantly, CTLs releasing IFN-γ against autologous tumour cells indicated that the neoepitope predicted by our pipeline was expressed on the surface of tumour cells in the context of HLA class I molecules. If the neoantigen elicits effective anti-tumour immune responses in the tumour microenvironment, the tumour should not grow in patients. As we stated above, because of several immune suppressive factors in the tumour microenvironment, a neoantigen may not be able to elicit an immune response. Eliciting an immune response against a tumour-specific neoantigen e*x vivo*, where we can eliminate immune suppressive factors by adoptive neoantigen-specific CTL therapy as an example, may be a useful strategy to enforce a neoantigen-specific response. Although we recognize the limitations of a single preclinical model, these results demonstrated the theoretical rationale for clinical application of neoantigen peptides.

Several limitations in this study should be stated. First, we predicted neoantigens only for each nonsynonymous SNV depending on the binding affinity for HLA class I. Because INDELs including gene fusions are reported to be good resources of highly immunogenic neoantigens^35,36^ and neoantigens with high affinity for HLA class II are reported to have an important role in the anti-tumour response^37,38^, these factors should be included in predicting neoantigens in future studies. Second, the proportion of tumour cells in the specimen probably affected the neoantigen load because a mutation was called for an mRNA read count relative to that of normal sequences. In the future, a neoantigen analysis system should be developed, which takes into account the tumour purity of specimens. Third, the number of specimens was small. Because breast cancers are reported to consist of multiple cancer subtypes^39^, a study should ideally be performed based on subtypes and a large number of specimens are needed for such analyses. Finally, we did not check whether the predicted neoantigen peptides were expressed on the tumour surface with MHC class I molecules and elicited specific CTLs in the tumour microenvironment. In pursuing the therapeutic application of neoantigen peptides, this may be an important direction to explore.

### Conclusions

We performed neoantigen analysis of fresh tumour specimens from breast cancer patients. The neoantigen load was not positively correlated to immune-related gene expression. However, synthesized neoantigen peptide-pulsed DCs induced CTLs from peripheral lymphocytes ex vivo. Collectively, these results indicate that endogenous neoantigens in the tumour microenvironment of breast cancer may be insufficient to elicit an immune response in the tumour microenvironment. Therefore, inducing neoantigen-specific immune responses outside the tumour microenvironment by adoptive neoantigen-specific CTLs or neoantigen-pulsed DC vaccines may be alternative strategies to use neoantigen prediction analysis.

## Methods

All methods in this study were approved by the Ethical Committees of Kyushu University Hospital (No. 128) and Fukuoka General Cancer Clinic (FGCC-EC001) based on the Act on Securement of Safety on Regenerative Medicine in Japan. This study complied with the Declaration of Helsinki and written informed consent was obtained from all patients.

### Breast cancer patients and samples

This study included 31 patients with histologically confirmed breast cancer from Kyushu University and Fukuoka General Cancer Clinic. Fresh breast cancer specimens were obtained immediately after biopsies or tumour resection and placed in microtubes that contained the RNA stabilization solution RNAlater (Thermo Fisher Scientific Inc., Tokyo, Japan). PBMCs were obtained by leukapheresis procedure using Haemonetics CCS (Haemonetics, MA, USA) in accordance with the manufacturer’s instructions. The leukapheresis product was diluted with RPMI-1640 medium (Kojin-Bio Inc., Saitama, Japan) for density gradient isolation on Ficoll-Hypaque. After isolation, the cells were washed three times with RPMI-1640 medium and the obtained PBMCs were cryopreserved at − 80 °C. Tumour specimens and PBMCs were obtained at various time points from patients after diagnosis (as shown in Table 1).

### Establishment of primary breast cancer cells from a patient with chemo-refractory metastatic triple negative breast cancer (TNBC)

Pleural fluid associated with carcinomatous pleurisy and PBMCs were obtained from a 33-year-old woman with chemo-refractory TNBC, who was diagnosed and treated at Kyushu University. Cytology of the pleural fluid revealed numerous tumour cells. The cells were first cultured in DMEM/F12 (Life Technologies, Grand Island, NY, USA). After formation of colonies, they were cultured in DMEM/F12 supplemented with 10% fetal bovine serum (Thermo Fisher Scientific, Waltham, MA, USA) and cryopreserved at − 80 °C. We have previously reported the phenotype of the established primary breast cancer cells (MUK-BC1)^40^.

### Whole exome sequencing and RNA sequencing (RNA-seq)

Genomic DNA and total RNA were extracted from fresh tumours in RNAlater using the AllPrep DNA/RNA mini kit (Qiagen Inc., Venlo, Netherlands) in accordance with the manufacturer’s instructions. Control genomic DNA was extracted from patient-matched PBMCs. Whole exome libraries were prepared from 200 to 3000 ng genomic DNA using the SureSelect Human All Exon V6 kit (Agilent Technologies Inc., Santa Clara, CA, USA) in accordance with the manufacturer’s instructions. RNA-seq libraries were prepared using the TruSeq Stranded mRNA Library Prep kit (Illumina Inc., San Diego, CA, USA). The prepared whole exome and RNA-seq libraries were sequenced by a HiSeq 4000 at 2 × 100 bp or NovaSeq 6000 sequencer at 2 × 150 bp (Illumina). To analyse RNA-seq data, we used Cufflinks software^41^ and FPKM (fragments per kilobase of transcript per million mapped reads) values were calculated for immune-related gene expression analysis.

### Read mapping and variant calling

Sequence alignment and mutation calling were performed as described previously^42^. For whole exome sequencing, the sequence reads were mapped to the human reference genome GRCh37/hg19 using Burrows–Wheeler Aligner (v0.7.10)^43^. Possible PCR duplicates, read pairs with a mapping quality of < 30, and mismatches of more than 5% were excluded. For RNA-seq, the sequence reads were mapped to the human reference genome GRCh37/hg19 using STAR (v2.4.0a)^44^. Somatic variants were called using Fisher’s exact test-based methods in accordance with the following parameters as described previously^42^: (1) base quality of ≥ 15; (2) sequence depth of ≥ 10; (3) variant depth of ≥ 4; (4) variant frequency in tumour of ≥ 10%; (5) variant frequency in normal samples of < 2%; (6) Fisher *P*-value of < 0.05. SNVs, indels, and splicing region were annotated using ANNOVAR^45^. We classified the somatic mutations as follows: (1) non-synonymous SNVs (nsSNVs); (2) splicing; (3) stop gain; (4) stop loss; (5) insertion/deletion (INDEL); (6) synonymous SNVs (sSNVs). Splicing mutation indicates that a single nucleotide variation occurred at the position of the splicing acceptor of the donor site.

We next used RNA-seq data of tumours to examine gene expression. The actual counts of sequence reads that covered the somatic mutated position were used to confirm mRNA expression of mutated genes^46^. This strategy was used, as if the FPKM value was used, the mRNA expression level would be calculated from the sum of mutated and non-mutated genes. The total RNA-seq read counts were > 20,000,000 per specimen.

### Prediction of HLA class I-binding peptides

On the basis of whole exome sequencing data from normal DNA of PBMCs, HLA class I genotypes (HLA-A, -B, and -C) were determined by the OptiType algorithm^47^. We used only nsSNVs derived from possible peptides to predict HLA genotype-restricted neoantigens because the number of INDELs and fusions, if detected by RNA-seq, was much lower than that of nsSNVs and our neoantigen prediction algorithm for INDELs/fusions was under development and not yet validated^42^. Briefly, we examined all possible 8–11-mer peptides that harboured each substituted amino acid by filtering with the predicted binding affinity to HLA-A, -B, and -C of IC_50_ < 500 nM using NetMHCv3.4 and NetMHCpanv2.8 software^48,49^.

In the selection of neoantigen epitopes, we also considered the mRNA expression level of mutated genes by counting RNA-seq reads that contained the nsSNVs.

### Generation of DCs from PBMCs

DCs were generated from cryopreserved PBMCs. PBMCs were thawed and cultured in 6-well plates (BD FALCON) that contained RPMI-1640 medium for 30 min. After removal of floating cells and washing the plate with RPMI-1640 medium, adherent cells were cultured in complete DC medium that consisted of RPMI-1640 medium with recombinant human granulocyte/monocyte colony-stimulating factor (GM-CSF; 100 ng/ml; Primmune Inc., Kobe, Japan) and recombinant human interleukin-4 (IL-4; 50 ng/ml; Primmune Inc.). On day 6, the cells were stimulated with a maturation cytokine cocktail that contained human recombinant tumour necrosis factor-α (TNF-α; 500 U/ml; PeproTech Inc., NJ, USA) and interferon-α (IFN-α; 500 U/ml; Dainippon Pharma, Osaka, Japan) for 18 h. Morphological changes were monitored by light microscopy and cells were examined by flow cytometric analysis. DCs that expressed high levels of HLA class I, HLA-DR, CD40, and a costimulatory molecule (CD86) with negative expression of CD14 (data not shown) were used for further experiments and referred to as mature DCs (mDCs).

### Generation of neoantigen peptide-pulsed cytotoxic T lymphocytes (CTLs) ex vivo

After pulsing mDCs with the indicated synthesized neoantigen peptides (Table 3, closed square) for 4 h, we cocultured peptide-pulsed mDCs with peripheral lymphocytes obtained by excluding plastic-adherent monocytes from PBMCs. RPMI-1640 medium that contained 10% healthy human serum and recombinant human interleukin-2 (IL-2; 40 U/ml; Primmune Inc.) was used as the culture medium. We exchanged the medium once every 3–4 days. After 2 weeks of coculture, we harvested the lymphocytes and cocultured them with newly generated peptide-pulsed mDCs for another 2 weeks under the same culture condition used for the first coculture. The lymphocytes were then harvested and used as CTLs for further experiments.

### ELISpot assay

The ELISpot assay was performed using the Human IFN-γ ELISpot^plus^ kit (MABTECH, Cincinnati, OH, USA) in accordance with the manufacturer’s instructions. Briefly, 96-well plates with nitrocellulose membranes (Millipore, Molshelm, France) precoated with a primary anti-IFN-γ antibody were pretreated with RPMI-1640 medium that containrf 10% autologous serum at 4 °C overnight. A total of 1 × 10^4^ autologous immature DCs were added to each well along with the DC maturation cocktail and the cells were incubated overnight. Synthesized neoantigen peptide (final concentrations of 5, 50, and 100 µg/ml) was added to each well and the cells were incubated for 4 h. After three washes with RPMI-1640 medium, 1.0 × 10^5^ or 1.5 × 10^5^ autologous peripheral lymphocytes were added to each well and the cells were incubated for 48 h. After three washes with PBS, the secondary antibody was added to each well and the cells were incubated for 2 h. The plates were incubated with HRP reagent and stained with TNB (MABTECH). Spots were captured and analysed by an automated ELISpot reader 08 classic (AID GmbH, Strasberg, Germany) and positivity of the neoantigen-specific T cell response was quantitatively defined as specific spots.

We also performed an ELISpot assay to examine the IFNγ-releasing ability of CTLs against autologous tumour. Briefly, target tumour cells (5 × 10^3^ per well) were seeded in ELISpot 96-well plates prepared as described above and cultured overnight. CTLs were then added to the wells (5 × 10^4^ per well) and the cells were incubated for 4 h at 37 °C, followed by the same procedure to detect IFN-γ spots as described above.

### Calcein-release cytotoxicity assay and cell imaging

Target tumour cells (5 × 10^3^ per well) were seeded in a 96-well flat-bottom plate and cultured overnight. The cells were then incubated at 37 °C for 1 h in the presence of calcein-AM. CTLs were then added to the wells (5 × 10^4^ per well) and the cells were incubated with tumour cells for 4 h at 37 °C. Images were captured under a conventional fluorescence microscope (IX81; Olympus, Tokyo, Japan) equipped with a colour CCD camera (DPI72; Olympus) and objective lens (LUC plan FLN; Olympus). All procedures were performed at 20–25 °C. Images were analysed using Lumina Vision software. The cytotoxicity percentage was calculated as (control fluorescence—sample fluorescence)/control × 100%.

### Statistical analyses

All data are expressed as the mean ± standard error of the mean (SEM). Differences between groups were assessed for statistical significance using the paired Student’s *t*-test. Pearson correlation (r) was used for correlation analysis between all examined parameters. A *p-*value of < 0.05 was considered statistically significant. Statistical analyses were performed using GraphPad Prism Version 8.3.0 (GraphPad software, La Jolla, CA, USA).

### Ethics approval and consent to participate

This study was approved by the Ethics Committees of Kyushu University Hospital (No. 128) and Fukuoka General Cancer Clinic (FGCC-EC001).

## Supplementary Information

Supplementary Information.

## Data Availability

The datasets used and/or analysed during the current study are available from the first or corresponding author on reasonable request.

## Abbreviations

CTL: Cytotoxic T lymphocyte
DC: Dendritic cell
FPKM: Fragments per kilobase of transcript per million mapped reads
ICI: Immune checkpoint inhibitor
INDEL: Insertion/deletion
nsSNV: Nonsynonymous single nucleotide variant
PBMC: Peripheral blood mononuclear cell
sSNV: Synonymous single nucleotide variant
TIL: Tumour-infiltrating lymphocyte
TMB: Tumour mutation burden
TNBC: Triple negative breast cancer
