# Supplementary material for: Neoantigens elicit T cell responses in breast cancer
Source: Sci Rep. 2021 Jun 30;11:13590. doi: 10.1038/s41598-021-91358-1 (PMC8245657; doi:10.1038/s41598-021-91358-1)
Supplement: Supplementary file 1 — Supplementary Information. [file 41598_2021_91358_MOESM1_ESM.pdf]

Neoantigens elicit T cell responses in breast cancer

Takafumi Morisaki[1,2†], Makoto Kubo[1,2*†], Masayo Umebayashi[2], Poh Yin Yew[3], Sachiko Yoshimura[3], Jae-Hyun Park[3], Kazuma Kiyotani[5], Masaya Kai[1], Mai Yamada[1], Yoshinao Oda[4], Yusuke Nakamura[5], Takashi Morisaki[2], and Masafumi Nakamura[1]

1. Department of Surgery and Oncology, Graduate School of Medical Sciences, Kyushu University, Fukuoka, Japan
2. Fukuoka General Cancer Clinic, Fukuoka, Japan
3. Cancer Precision Medicine, Inc., Kawasaki, Kanagawa, Japan.
4. Department of Anatomic Pathology, Graduate School of Medical Sciences, Kyushu University, Fukuoka, Japan
5. Cancer Precision Medicine Center, Japanese Foundation for Cancer Research, Tokyo, Japan

Supplementary Information

Correlation analysis between the number of neoantigens (IC_{50} < 500 nM, read count ≥ 1) and immune-related gene expression (FPKM).

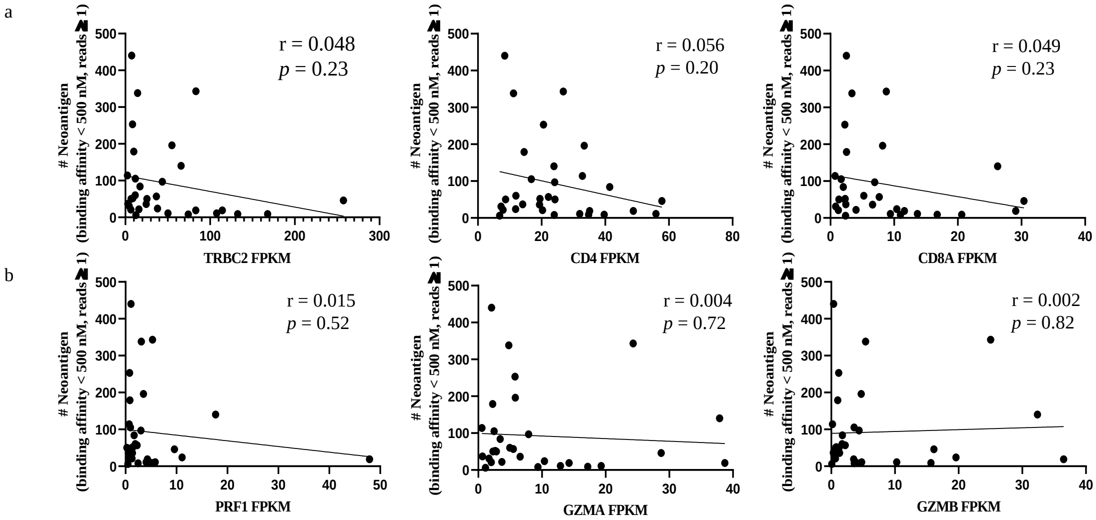

a. The number of neoantigens was not correlated to expression of T cell marker genes (*TRBC*, *CD4*, and *CD8*). **b.** The number of neoantigens was not correlated to expression of T cell activation marker genes (*PRF1*, *GZMA*, and *GZMB*).
